# Norsesquiterpenoids from the leaves of *Croton tiglium*

**DOI:** 10.1007/s13659-011-0035-3

**Published:** 2012-01-02

**Authors:** Wei Bu, Yan-Ni Shi, Yong-Ming Yan, Qing Lu, Guang-Ming Liu, Yan Li, Yong-Xian Cheng

**Affiliations:** 1 State Key Laboratory of Phytochemistry and Plant Resources in West China, Kunming Institute of Botany, Chinese Academy of Sciences, Kunming, 650201 China; 2 Graduate University of Chinese Academy of Sciences, Beijing, 100049 China; 3 Faculty of Pharmacy, Dali University, Dali, 671000 China

**Keywords:** *Croton tiglium*, badounoid, norsesquiterpenoid, cytostatic activity

## Abstract

Two new compounds, badounoids A (**1**) and B (**2**), together with 13 known norsesquiterpenes, were isolated from the leaves of *Croton tiglium* L. The structures of the new compounds were established by means of spectroscopic methods. The absolute configuration of badounoid B was determined by single-crystal X-ray diffraction analysis. All the known compounds were isolated from *Croton* plants for the first time which added a new chemical facet for this genus. The selected compounds were evaluated for their cytostatic activity against several cancer cell lines. None of them was found to be active.

**Electronic Supplementary Material:**

Supplementary material is available for this article at 10.1007/s13659-011-0035-3 and is accessible for authorized users.

## Introduction

The intriguing structures of Euphorbiaceae plants and their diverse biological activities have attracted great interest in the recent years.[1] *Croton tiglium* L. is a plant belonging to the family Euphorbiaceae, its seeds, a well-known traditional Chinese medicine have been extensively investigated. So far, diterpenoids, alkaloids, flavonoids, and steroids have been characterized from the seeds, they were found to have antitumor, antiinflammatory, analgesic, and lipid lowering effects.[2] The leaves of *C. tiglium* have been used to treat diarrhea, tinea, pain, and hurt,[3] however, little is known for its chemical profiling. During our search for active compounds from the leaves, fifteen norsesquiterpenes including two new ones were isolated and structurally identified. This paper describes their isolation and structural identification.

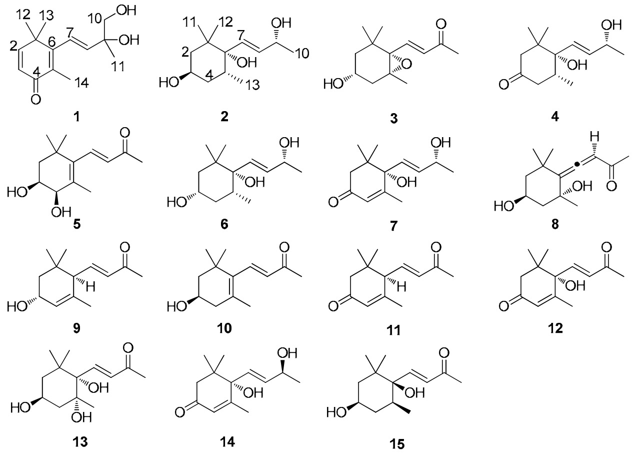

## Results and Discussion

Badounoid A (**1**), isolated as colorless gums, had the molecular formula $C_{14}H_{20}O_3$ derived from its positive HRESIMS at *m/z* 259.1318 $[M + Na]^+$ (calcd. 259.1310), indicating five degrees of unsaturation. The IR spectrum showed the absorption bands for hydroxy (3431 $cm^{-1}$) and $\alpha,\beta$-unsaturated carbonyl (1654 $cm^{-1}$) groups. The $^{13}C$ NMR and DEPT spectra revealed 14 carbon resonances, which are four methyl, one oxygenated methylene, four methine, and five quaternary carbons (including one oxygenated carbon and one carbonyl), indicating that **1** is an analogue of **5**. The $^1H$-$^1H$ COSY correlation of H-2 ($\delta$ 6.99)/H-3 ($\delta$ 6.18), and HMBC correlations of H-2, H-3, Me-14/C-4 ($\delta$ 188.9), Me-14/C-5 ($\delta$ 131.3), C-6 ($\delta$ 161.9), and Me-12/C-1 ($\delta$ 41.0), C-6 (Figure 1) suggested the west part of **1** as shown. The side chain of **1** was identified as a substituted isoprenyl group according to the following evidence: (i) $^1H$-$^1H$ COSY correlation of H-7 ($\delta$ 6.43)/H-8 ($\delta$ 5.91), (ii) HMBC correlations of H-8, H-10 ($\delta$ 3.49), Me-11 ($\delta$ 1.34)/C-9 ($\delta$ 74.6). Further, HMBC correlations of H-7, H-8/C-6 established the linkage of the side chain with the ring. The $J_{H-7,H-8}$ value of 16.3 Hz indicated a *trans* double

*To whom correspondence should be addressed. E-mail: yxcheng@mail.kib.ac.cn

**Table 1. NMR data for compounds 1 and 2.[a] (methanol-$d_4$ for 1 and $CDCl_3$ for 2, *J* in Hz, δ in ppm)**

| position | **1** $\delta_C$ | **1** $\delta_H$ | **2** $\delta_C$ | **2** $\delta_H$ |
|---|---|---|---|---|
| 1 | 41.0, C | | 37.4, C | |
| 2 | 160.0, CH | 6.99 (d, 9.9) | 42.2, $CH_2$ | 1.38 (m); 1.82 (dd, 14.6, 3.4) |
| 3 | 126.2, CH | 6.18 (d, 9.9) | 67.3, CH | 4.11 (m) |
| 4 | 188.9, C | | 36.4, $CH_2$ | 1.55 (m); 1.69 (m) |
| 5 | 131.3, C | | 29.1, CH | 2.26 (m) |
| 6 | 161.9, C | | 77.9, C | |
| 7 | 125.0, CH | 6.43 (d, 16.3) | 133.5, CH | 5.67 (d, 15.8) |
| 8 | 144.2, CH | 5.91 (d, 16.3) | 133.7, CH | 5.74 (dd, 15.8, 5.1) |
| 9 | 74.6, C | | 68.6, CH | 4.38 (m) |
| 10 | 70.7, $CH_2$ | 3.49 (s) | 23.8, $CH_3$ | 1.29 (d, 6.4) |
| 11 | 24.7, $CH_3$ | 1.34 (s) | 27.1, $CH_3$ | 1.15 (s) |
| 12 | 27.0, $CH_3$ | 1.27 (s) | 25.6, $CH_3$ | 0.82 (s) |
| 13 | 26.9, $CH_3$ | 1.27 (s) | 15.8, $CH_3$ | 0.75 (d, 6.8) |
| 14 | 13.3, $CH_3$ | 1.94 (s) | | |

[a]Data were recorded at 500 MHz for $^1$H NMR and 125 MHz for $^{13}$C NMR.

bond. The configuration at C-9 still remained unresolved, since the stereochemistry determination at the chiral center of the conformationally flexible chain is always challengeable. Thus, the structure of **1** was deduced as shown, with a trivial name badounoid A.

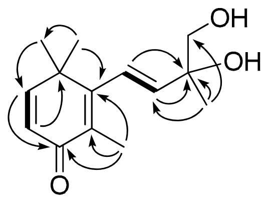

**Figure 1.** Selected HMBC (H→C) and COSY(—) correlations of **1**.

Badounoid B (**2**) was isolated as colorless crystals. The molecular formula of **2** was determined to be $C_{13}H_{24}O_3$ from its HRESIMS at *m/z* 227.1652 $[M - H]^-$ (calcd. 227.1647), requiring two degrees of unsaturation. The IR spectrum displayed the existence of OH (3430 cm$^{-1}$) and C=C (1638 cm$^{-1}$) functionalities. The NMR data of **2** resembled those of **6**. Interpretation of $^1$H-$^1$H COSY, HSQC and HMBC spectra of **2** disclosed that compounds **2** and **6** have the same planar structure. The ROESY correlation of H-5/H-7 suggested that Me-13 and OH-6 were spacially vicinal. Whereas, the scarcity of diagnostic ROESY signals made it difficult to assign the relative configuration at C-3. Thus, the configurations at C-3 and C-9 of flexible side chain were clarified by X-ray diffraction using Cu-irradiation (Figure 2), which also allowed the assignment of absolute configuration in **2** as 3*R*, 5*R*, 6*S*, and 9*R*. Therefore, the structure of **2** was determined as shown and given a name badounoid B.

The known compounds were identified as 3β-hydroxy-5α,6α-epoxy-7-megastigmen-9-one (**3**),[4] 4,5-dihydroblumenol A (**4**),[5] (3*S*,4*R*)-3,4-dihydroxy-β-ionone (**5**),[6] (3*S*,5*R*,6*S*,7*E*,9*R*)-3,6-dihydroxy-5,6-dihydro-β-ionol (**6**),[7] blumenol A (**7**),[5] grasshopper ketone (**8**),[8] (3*R*,6*R*,7*E*)-3-hydroxy-4,7-megastigmadien-9-one (**9**),[9] (+)-3-hydroxy-β-ionone (**10**),[10] (6*R*,7*E*)-4,7-megastigmadien-3,9-dione (**11**),[10] (*S*)-(+)-de-hydrovomifoliol (**12**),[11] (3*S*,5*R*,6*S*,7*E*)-3,5,6-trihydroxy-7-megastigmen-9-one (**13**),[12] corchoionol C (**14**),[13] and (+)-boscialin (**15**),[14] respectively, by comparison with literature data. All these compounds were isolated from this genus for the first time.

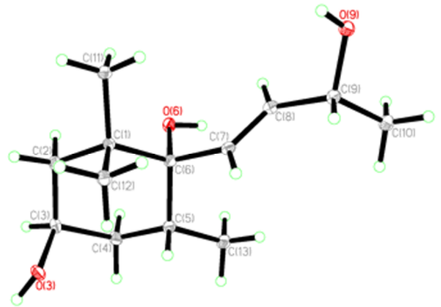

**Figure 2.** X-ray crystallographic structure of **2** showing the absolute configuration.

Megastigmane norsesquiterpenoids have been widely found in the plants. However, their real role in the plants or in drug discovery is poorly known. It was reported that this type of norsesquiterpene possesses antiinflammatory activity. Whether the present isolates being also responsible for the traditional uses of the leaves in infectious diseases needs further investigation. In this study, the selected compounds (**1**, **3**, **4**, **12**–**14**) were evaluated for their cytostatic activity against HL-60, SMMC-7721, A-549, MCF-7, and SW480 human cancer cells, however, all these compounds showed no activity in this assay.

Among these miscellaneous compounds, we noted that the position of OH may be at C-3, C-4, C-5, C-6, or C-9.The OH group at C-3 is readily oxidized into a ketone when a double bond is formed between C-4 and C-5. Likewise, the OH-3 tends to be eliminated when a ketone occurs at C-4. In this sense, we could tentatively conclude that compounds **5**, **8**, **9**, and **13** are probably unstable when they are exposed at oxidative environment.

## Experimental Section

**General Experimental Procedures**. Optical rotations were measured with a Jasco P-1020 polarimeter. IR spectra were obtained on a Tensor 27 with KBr pellets. UV spectra were measured on a Shimadzu UV-2401A spectrophotometer. NMR spectra were run on a DRX-500 MHz spectrometer with TMS as an internal standard. ESI and HRESIMS were determined with Auto Spec-3000 spectrometer. Silica gel (200–300 mesh, Qingdao Marine Chemical Co. Ltd., Qingdao, China), RP-18 gel (40–63 μm; Daiso Co., Osaka, Japan), and Sephadex

LH-20 (Amersham Biosciences, Uppsala, Sweden) were used for column chromatography (CC).

**Plant Material.** The leaves of *C. tiglium* were collected from Hubei Province, China, in July 2009. The material was identified by Prof. X. Zhou at Hunan University of Chinese Medicine. A voucher specimen (CHYX-0488) is deposited at the State Key Laboratory of Phytochemistry and Plant Resources in West China of our institute.

**Extraction and Isolation.** The air dried and powdered leaves of *C. tiglium* (75 Kg) were extracted with 80% EtOH (3 × 100 L) at 60°C and concentrated to give a crude extract (22 Kg), which was suspended in $H_2O$ and passed through a D101 macroporous resin column eluting with gradient aqueous EtOH. The 30% aq. EtOH portion (2 Kg) was fractionated by a silica gel CC eluted with $CHCl_3$ with increasing amounts of MeOH to afford fractions 1–3. The 50% aq. EtOH portion (2 Kg) was divided on silica gel eluted with gradient $CHCl_3$/MeOH (100:1–1:1) to afford fractions 4–7. The 70% aq. EtOH portion (2 Kg) was submitted to a silica gel CC eluted with gradient $CHCl_3$/EtOAc to afford fractions 8–10. Fraction 1 (280 g) was separated on a silica gel CC eluted with $CHCl_3$/$Me_2CO$ (15:1 to 5:1) and then subjected to Sephadex LH-20 (MeOH) to yield **3** (50 mg), **4** (10 mg) and **5** (6 mg). Fraction 2 (460 g) was submitted to a silica gel CC eluted with EtOAc/$Me_2CO$ (20:1 to 5:1) followed by RP-18 CC (MeOH/$H_2O$, 30:70 to 80:20) to yield **6** (57 mg), **2** (18 mg), **7** (10 mg) and **8** (10 mg). Fraction 4 (350 g) was separated by silica gel CC eluted with $CHCl_3$/$Me_2CO$ (10:1 to 5:1) followed by RP-18 CC (MeOH/$H_2O$, 20:70 to 80:20) and final purification by TLC ($CHCl_3$/*i*-PrOH, 15:1) to yield **9** (13 mg), **10** (8 mg), **11** (6 mg) and **12** (23 mg). Fraction 6 (420 g) was fractionated by silica gel CC eluted with $CHCl_3$/$Me_2CO$ (10:1 to 4:1) followed by RP-18 CC (MeOH/$H_2O$, 20:70 to 80:20) and final purification by Sephadex LH-20 (MeOH) to afford **13** (17 mg), **1** (21 mg) and **14** (11 mg). Fraction 9 (320 g) was separated on silica gel CC eluted with $CHCl_3$/EtOAc (5:1 to 1:1) and then Sephadex LH-20 (MeOH/$CHCl_3$, 6:4) to yield **15** (16 mg).

**Badounoid A (1):** colorless gum; $[\alpha]_D^{18}$ −1.4 (*c* 0.20 MeOH); UV (MeOH) $\lambda_{max}$ (log $\varepsilon$): 282 (3.28), 235 (3.42); IR (KBr) $\nu_{max}$ 3431, 1654, 1624 $cm^{-1}$; $^1H$ and $^{13}C$ NMR data, see Table 1; ESIMS *m/z* 259 $[M + Na]^+$; HRESIMS *m/z* 259.1318 $[M + Na]^+$ (calcd. for $C_{14}H_{20}O_3Na$ $[M + Na]^+$, 259.1310).

**Badounoid B (2):** colorless crystal; $[\alpha]_D^{18}$ −28.9 (*c* 0.23 MeOH); UV (MeOH) $\lambda_{max}$ (log $\varepsilon$): 201 (2.74); IR (KBr) $\nu_{max}$ 3430, 2925, 1638 $cm^{-1}$; $^1H$ and $^{13}C$ NMR data, see Table 1; ESIMS *m/z* 227 $[M - H]^-$; HRESIMS *m/z* 227.1652 $[M - H]^-$ (calcd. for $C_{13}H_{23}O_3$ $[M - H]^-$, 227.1647).

**Crystallographic Data for Compound 2:** $C_{13}H_{24}O_3$, $Mr$ = 204, Orthorhombic, space group $P2_12_12_1$, $a$ = 7.57300(10) Å, $b$ = 11.1050(2) Å, $c$ = 16.5082(3) Å, $V$ = 1388.31(4) Å$^3$, $Z$ = 4, $D_{calcd}$ = 1.179 g $cm^{-3}$, crystal size 0.33×0.20×0.08 $mm^3$, $F$ (000) = 544. The final $R_1$ value is 0.0375 ($wR_2$ = 0.1048) for 7580 reflections [I > 2σ(I)]. Flack structure parameter 0.2(2).

The crystallographic data for compound **2** has been deposited with the Cambridge Crystallographic Data Centre (deposit number CCDC 854477). Copies of the data can be obtained, free of charge, on application to the Director, CCDC, 12 Union Road, Cambridge CB2 1EZ, UK (fax: +44-(0)1223-336033) or email: deposite@ccdc.cam.ac.uk.

**Cytostatic Assay.** The cytostatic assay was performed using the MTT method, as previous method with slight modification.[15,16] Briefly, human tumor cells were seeded into 96-well plates and permitted to adhere for 12 h before drug addition. For suspended cells, they were seeded immediately before drug addition with an initial density of 1–2 × $10^5$ cells/mL. Each cell line was incubated with different concentrations of the compounds for 48 h. DDP and taxol were used as positive controls. Cell viability was measured and $IC_{50}$ values were calculated.

## Electronic Supplementary Material

Supplementary material is available in the online version of this article at http://dx.doi.org/ 10.1007/s13659-011-0035-3 and is accessible for authorized users.

## Acknowledgments

This work was financially supported by the Project of Natural Compound Library Construction from Chinese Academy of Sciences (KSCX2-EW-R-15).

**Open Access** This article is distributed under the terms of the Creative Commons Attribution License which permits any use, distribution, and reproduction in any medium, provided the original author(s) and source are credited.

## Electronic supplementary material

Supplementary material, approximately 4.27 MB.
