# Supplementary material for: Norsesquiterpenoids from the leaves of Croton tiglium
Source: Nat Prod Bioprospect. 2012 Jan 2;1(3):134–7. doi: 10.1007/s13659-011-0035-3 (PMC4131640; doi:10.1007/s13659-011-0035-3)
Supplement: Supplementary file 1 — Supplementary material, approximately 4.27 MB. [file 13659_2011_35_MOESM1_ESM.pdf]

Electronic Supplementary Material

Norsesquiterpenoids from the leaves of *Croton tiglium*

Wei BU,[a,c,†] Yan-Ni SHI,[a,b,†] Yong-Ming YAN,[a] Qing LU,[a] Guang-Ming LIU,[c] Yan LI,[a,*] and Yong-Xian CHENG[a,*]

[a]State Key Laboratory of Phytochemistry and Plant Resources in West China, Kunming Institute of Botany, Chinese Academy of Sciences, Kunming 650201, China

[b]Graduate University of Chinese Academy of Sciences, Beijing 100049, China

[c]Faculty of Pharmacy, Dali University, Dali 671000, China

[†]These authors contributed equally to this work.

Received 21 November 2011; Accepted 14 December 2011

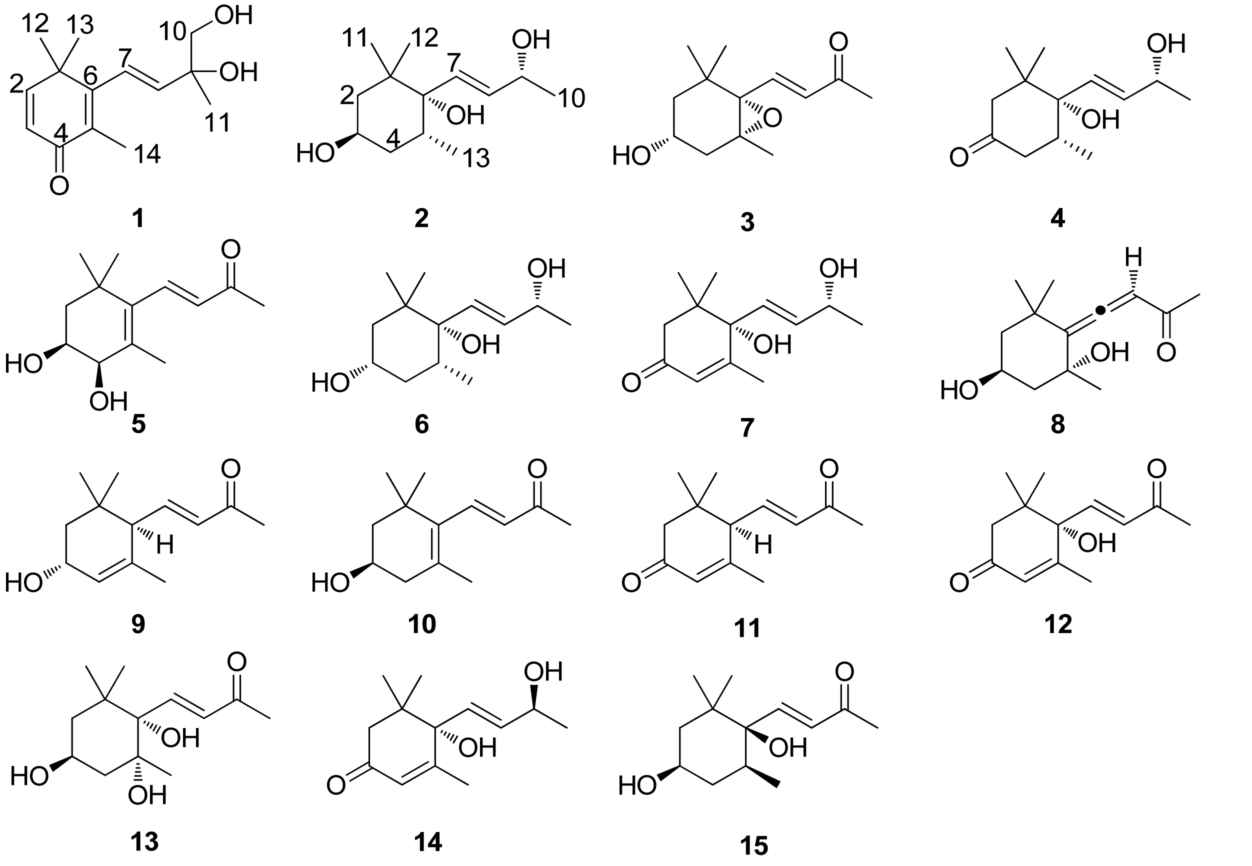

Structures of compounds **1**–**15**.

*To whom correspondence should be addressed. E-mail: yxcheng@mail.kib.ac.cn

Table of Contents

Figure 1. ^{1}H NMR spectrum of badounoid A (**1**)
Figure 2. ^{13}C NMR spectrum of badounoid A (**1**)
Figure 3. HSQC spectrum of badounoid A (**1**)
Figure 4. HMBC spectrum of badounoid A (**1**)
Figure 5. COSY spectrum of badounoid A (**1**)
Figure 6. ROESY spectrum of badounoid A (**1**)
Figure 7. ESI-MS spectrum of badounoid A (**1**)
Figure 8. HRESI-MS spectrum of badounoid A (**1**)
Figure 9. IR spectrum of badounoid A (**1**)
Figure 10. UV spectrum of badounoid A (**1**)
Figure 11. ^{1}H NMR spectrum of badounoid B (**2**)
Figure 12. ^{13}C NMR spectrum of badounoid B (**2**)
Figure 13. HSQC spectrum of badounoid B (**2**)
Figure 14. HMBC spectrum of badounoid B (**2**)
Figure 15. COSY spectrum of badounoid B (**2**)
Figure 16. ROESY spectrum of badounoid B (**2**)
Figure 17. ESI-MS spectrum of badounoid B (**2**)
Figure 18. HRESI-MS spectrum of badounoid B (**2**)
Figure 19. IR spectrum of badounoid B (**2**)
Figure 20. UV spectrum of badounoid B (**2**)

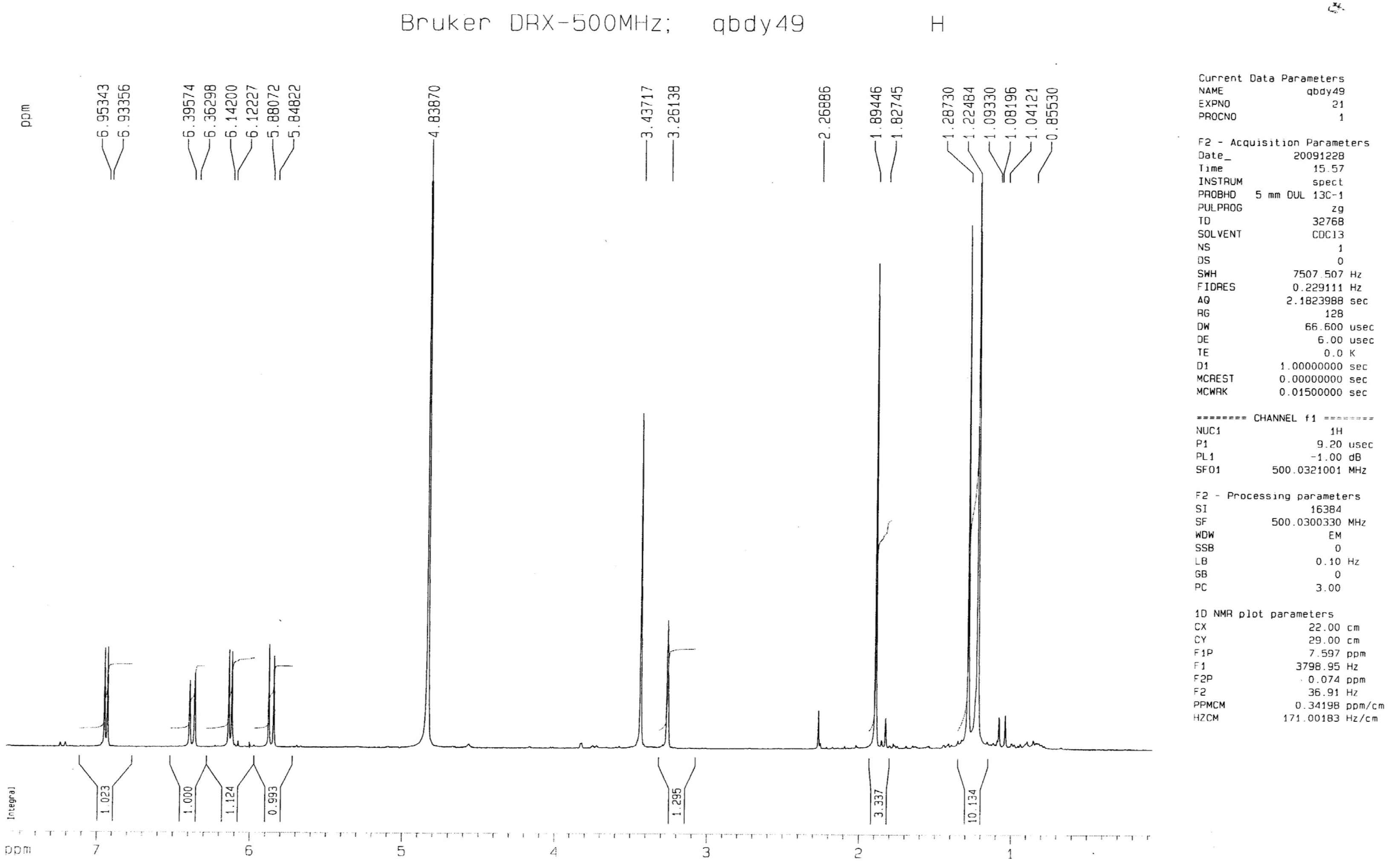

Figure 1. ^{1}H NMR spectrum of badounoid A (**1**)

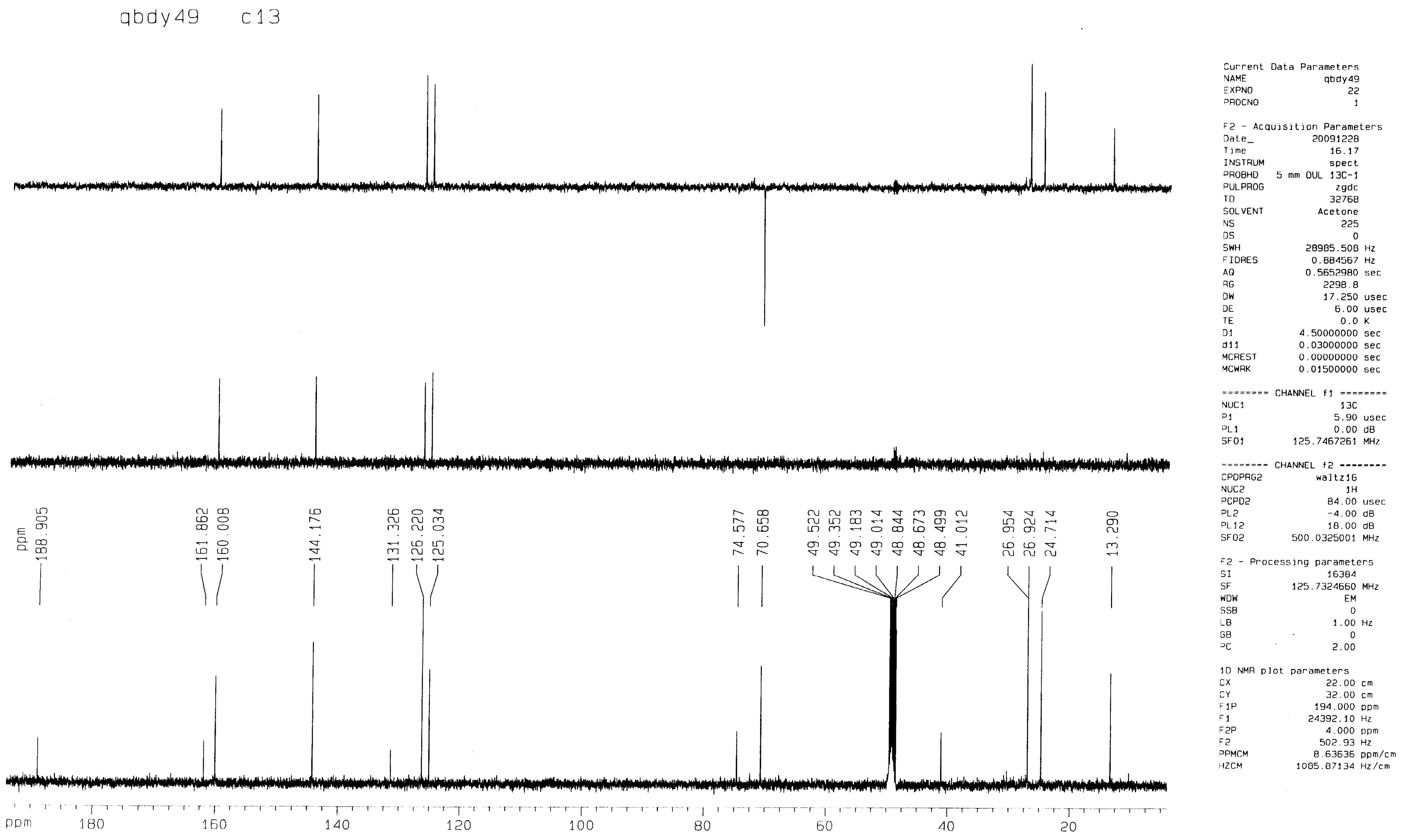

Figure 2. ^{13}C NMR spectrum of badounoid A (**1**)

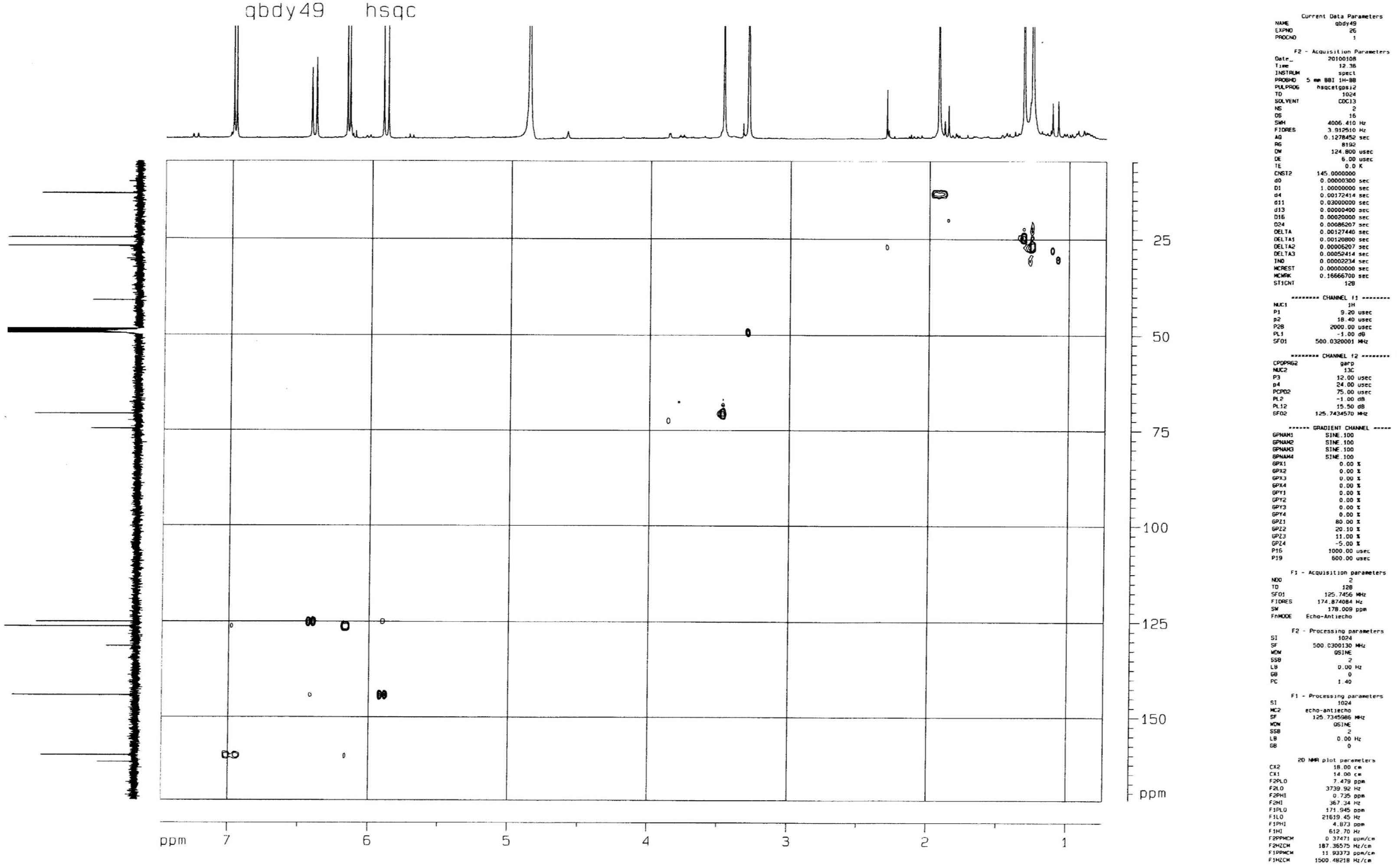

Figure 3. HSQC spectrum of badounoid A (**1**)

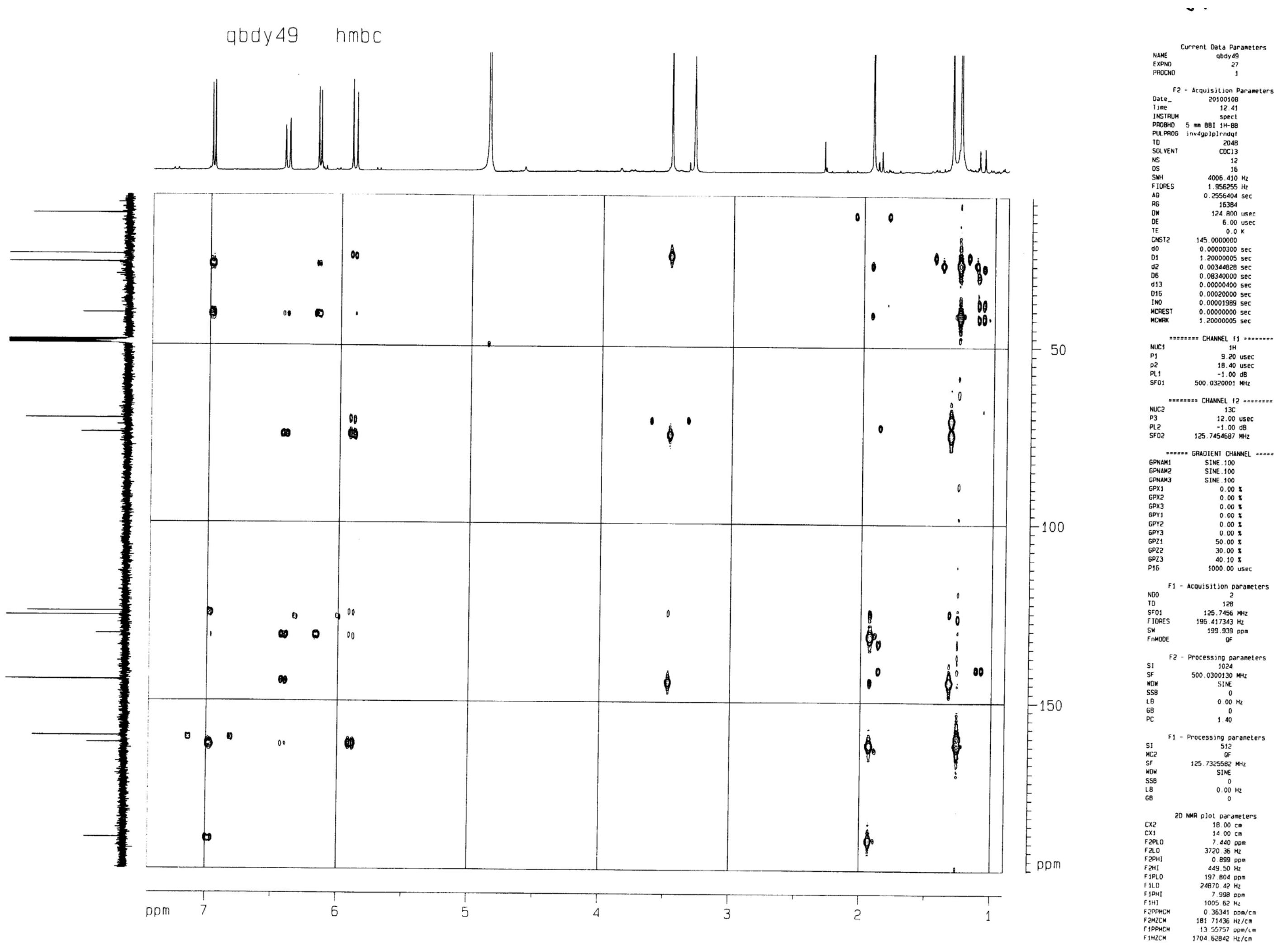

Figure 4. HMBC spectrum of badounoid A (**1**)

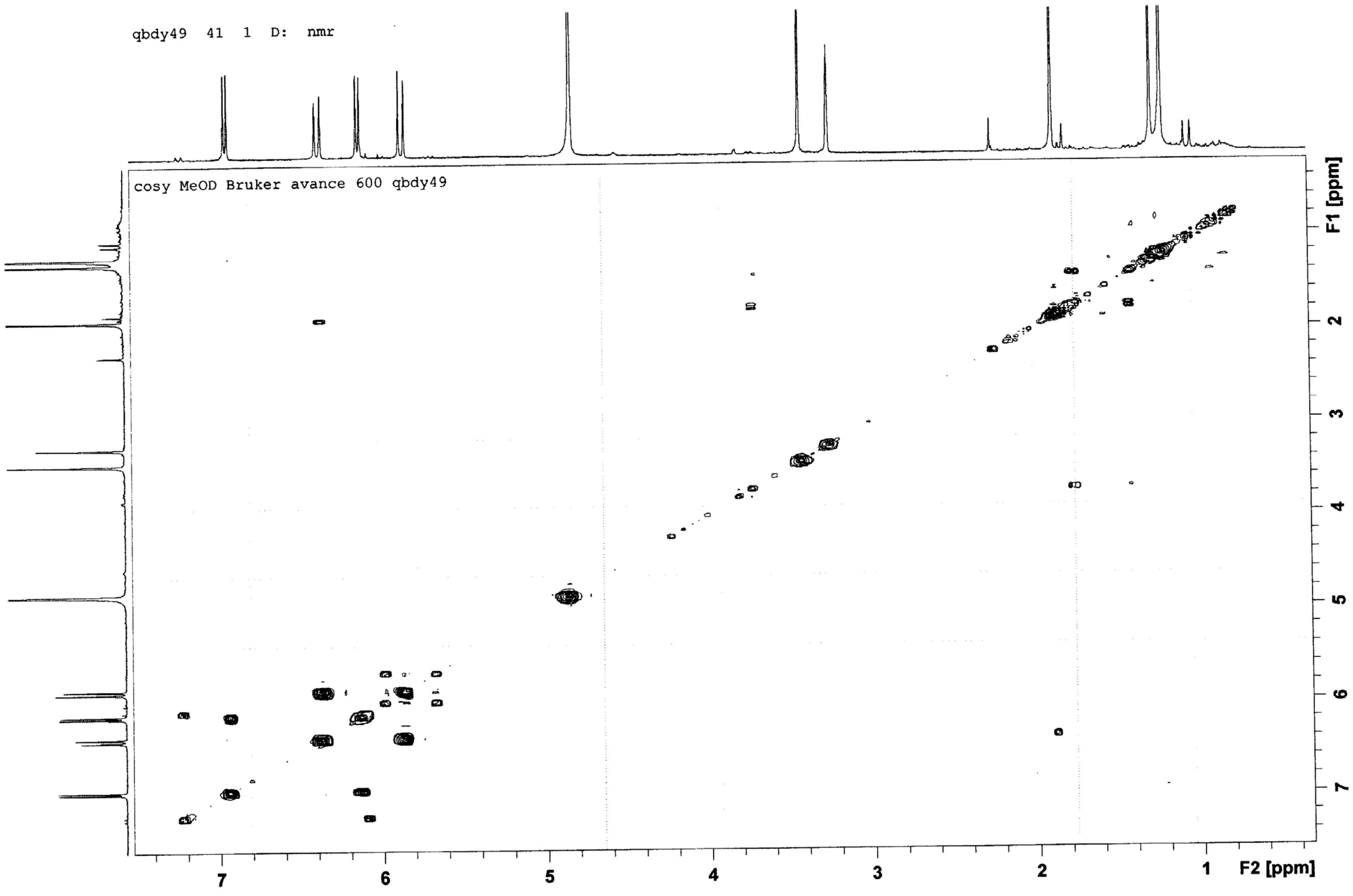

Figure 5. COSY spectrum of badounoid A (**1**)

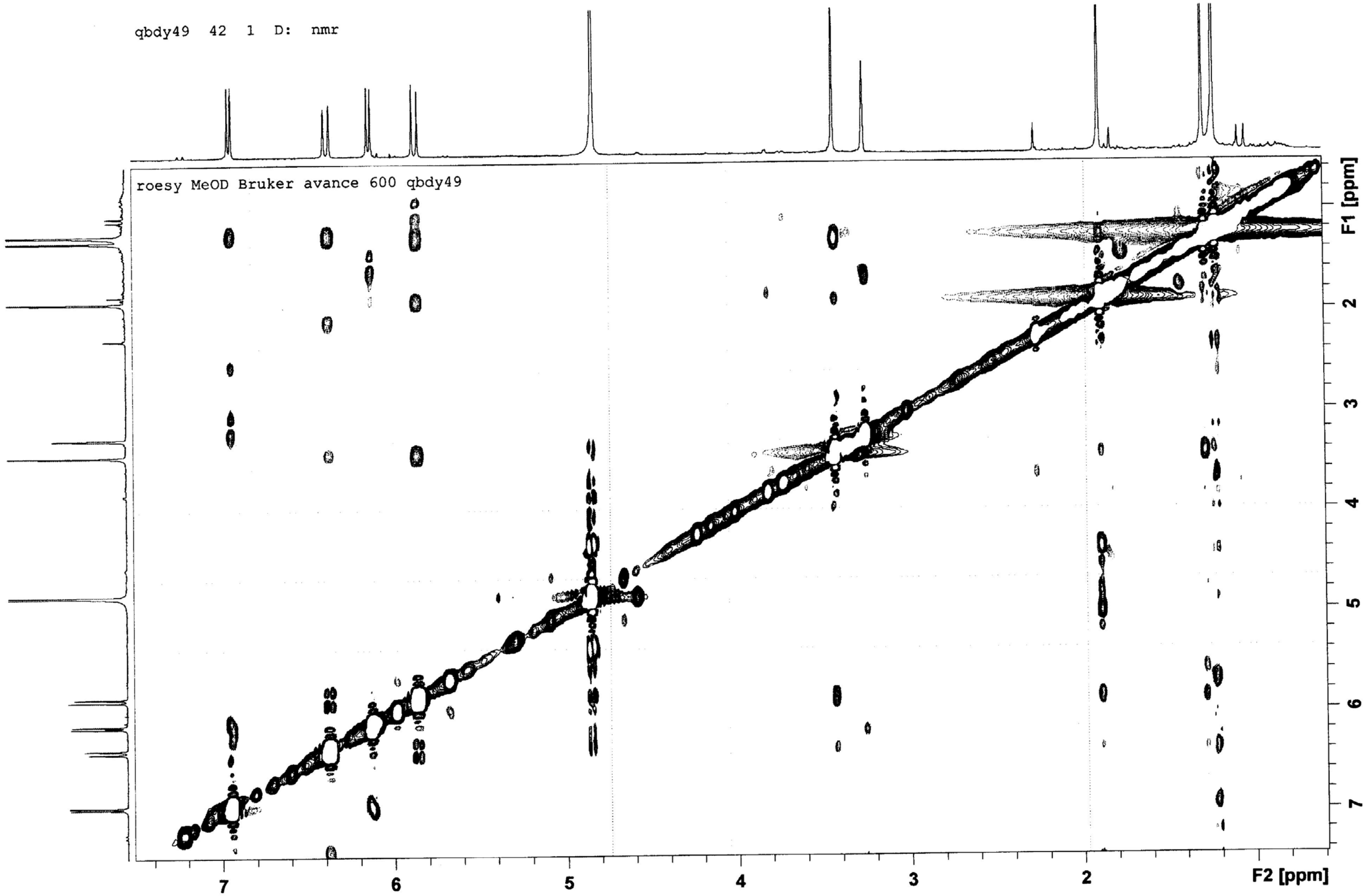

Figure 6. ROESY spectrum of badounoid A (**1**)

Acq. Date: Friday, April 16, 2010 Acq. Time: 13:09

Sample Name: 100416ESIN BDY-49

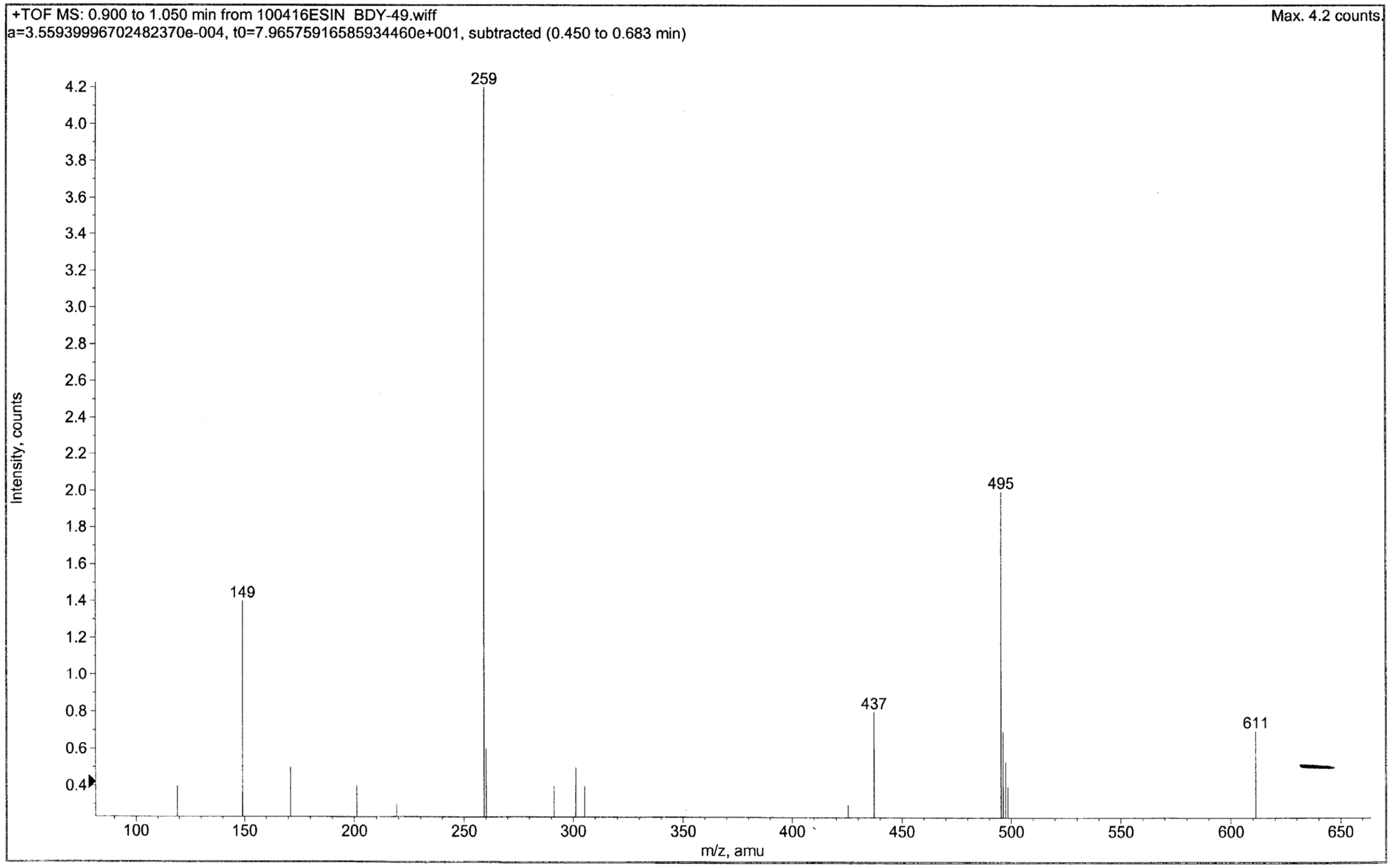

Figure 7. ESI-MS spectrum of badounoid A (**1**)

Acq. Date: Tuesday, November 15, 2011 Acq. Time: 13:04

Sample Name: 111115ESIA QBDY-49

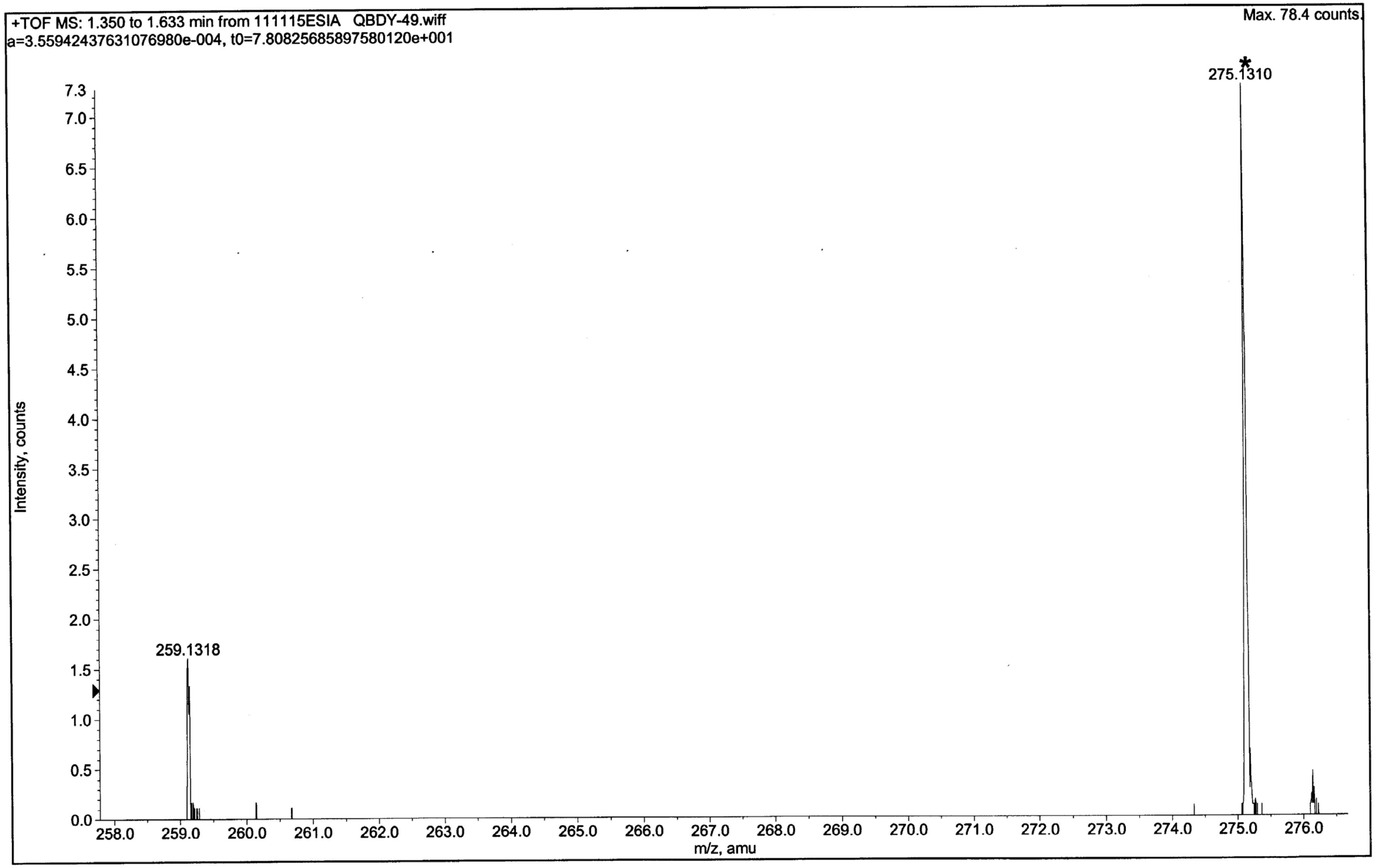

Figure 8. HRESI-MS spectrum of badounoid A (**1**)

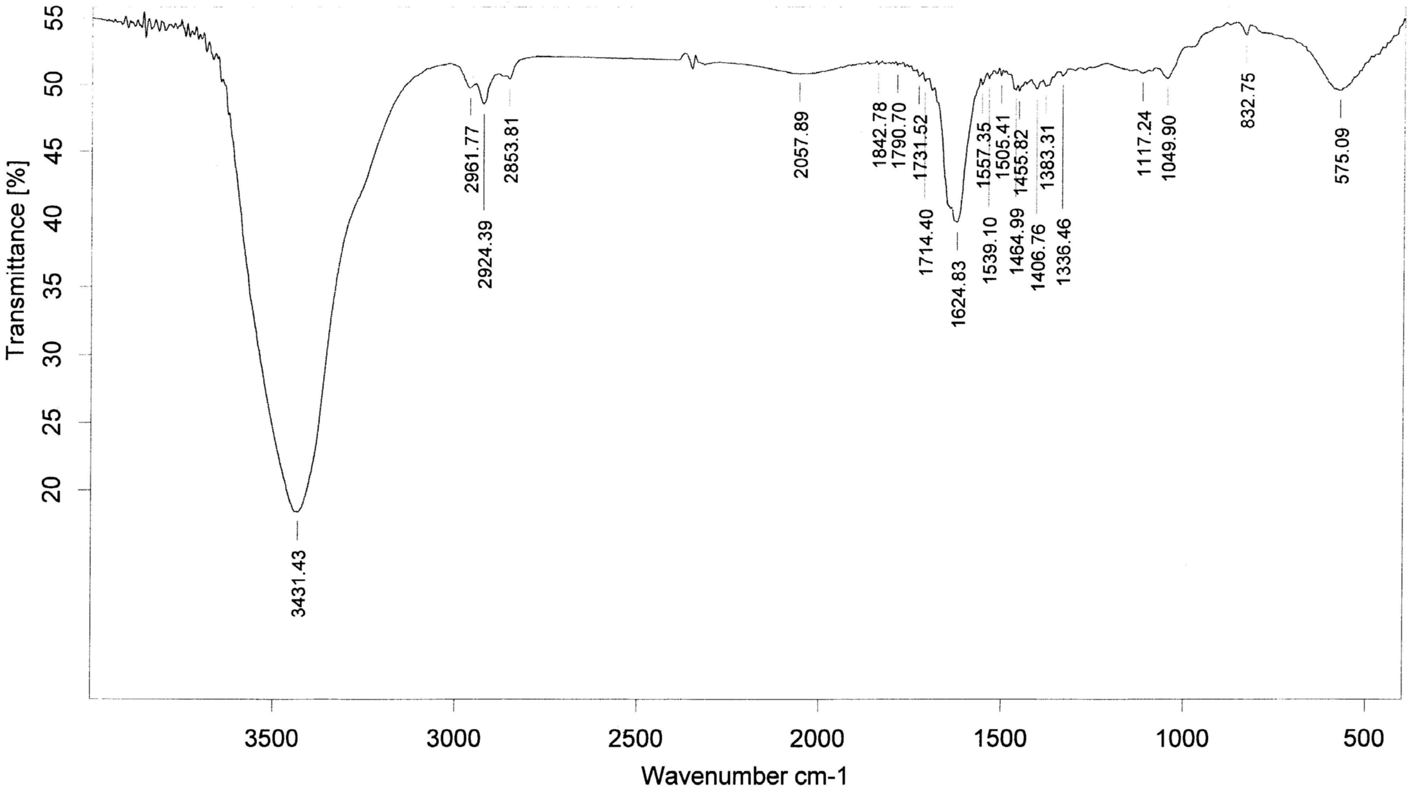

Sample : QBDY-49	Frequency Range : 399.246 - 3996.32	Measured on : 17/11/2011	
Technique : KBr压片	Resolution : 4	Instrument : Tensor27	Sample Scans : 16
Customer : 111117IR0	Zerofilling : 2	Acquisition : Double Sided,For	

Figure 9. IR spectrum of badounoid A (**1**)

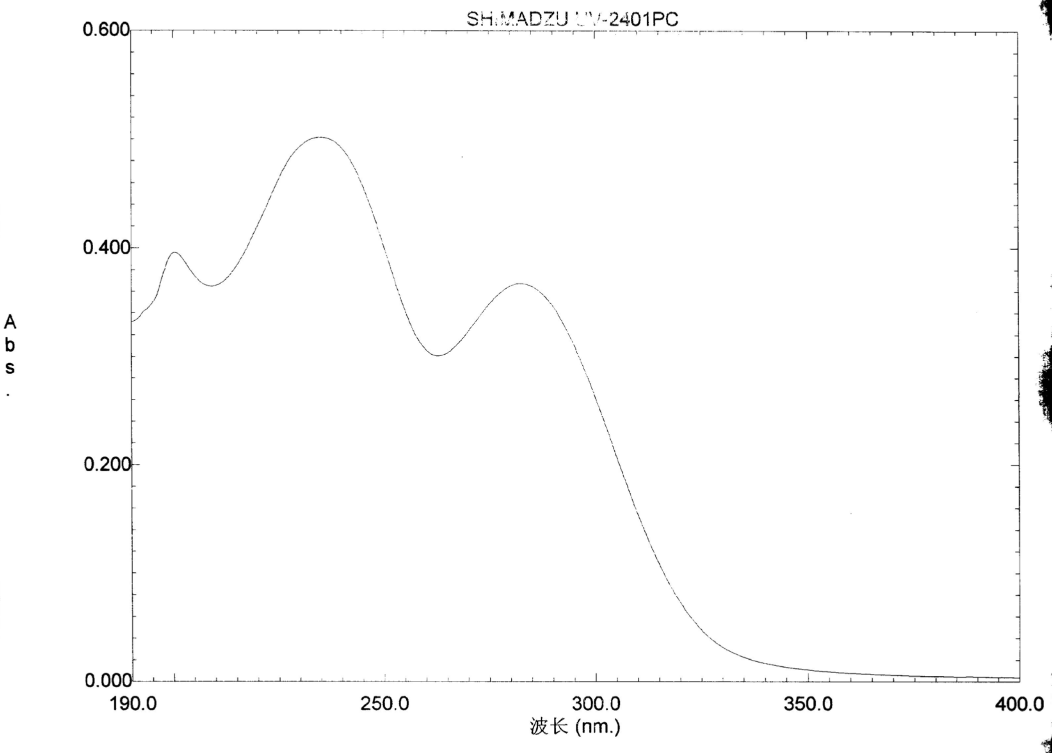

文件名: QBDY-49

QBDY-49

样品浓度：0.0090毫克/毫升
溶剂：甲醇

创建于: 01:03 11-07-28
数据: 原始

测量模式: Abs.
扫描速度: 中速
狭缝: 5.0
采样间隔: 0.2

否.	波长 (nm.)	Abs.
1	282.40	0.3673
2	235.00	0.5013
3	200.40	0.3957

Figure 10. UV spectrum of badounoid A (**1**)

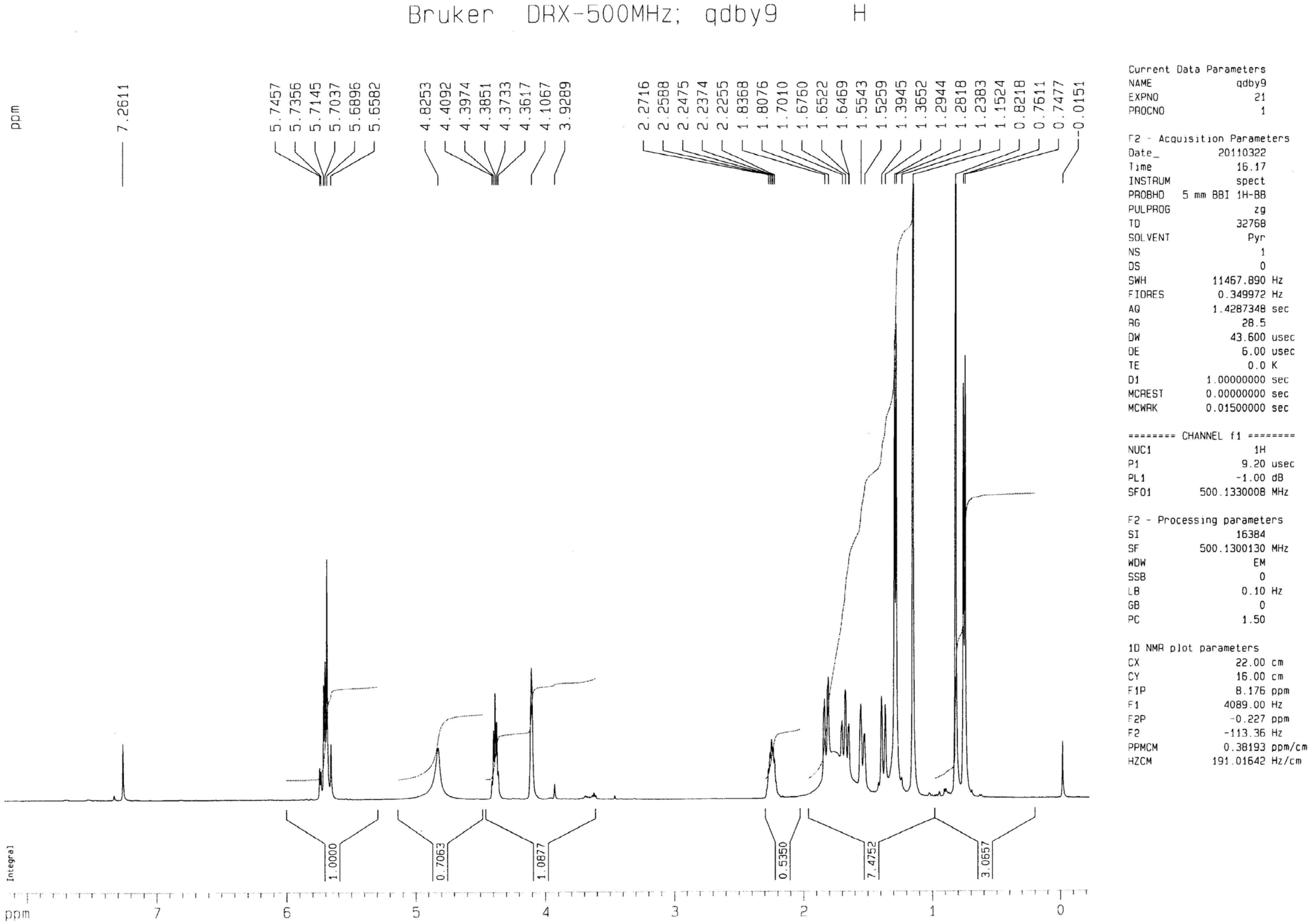

Figure 11. ^{1}H NMR spectrum of badounoid B (**2**)

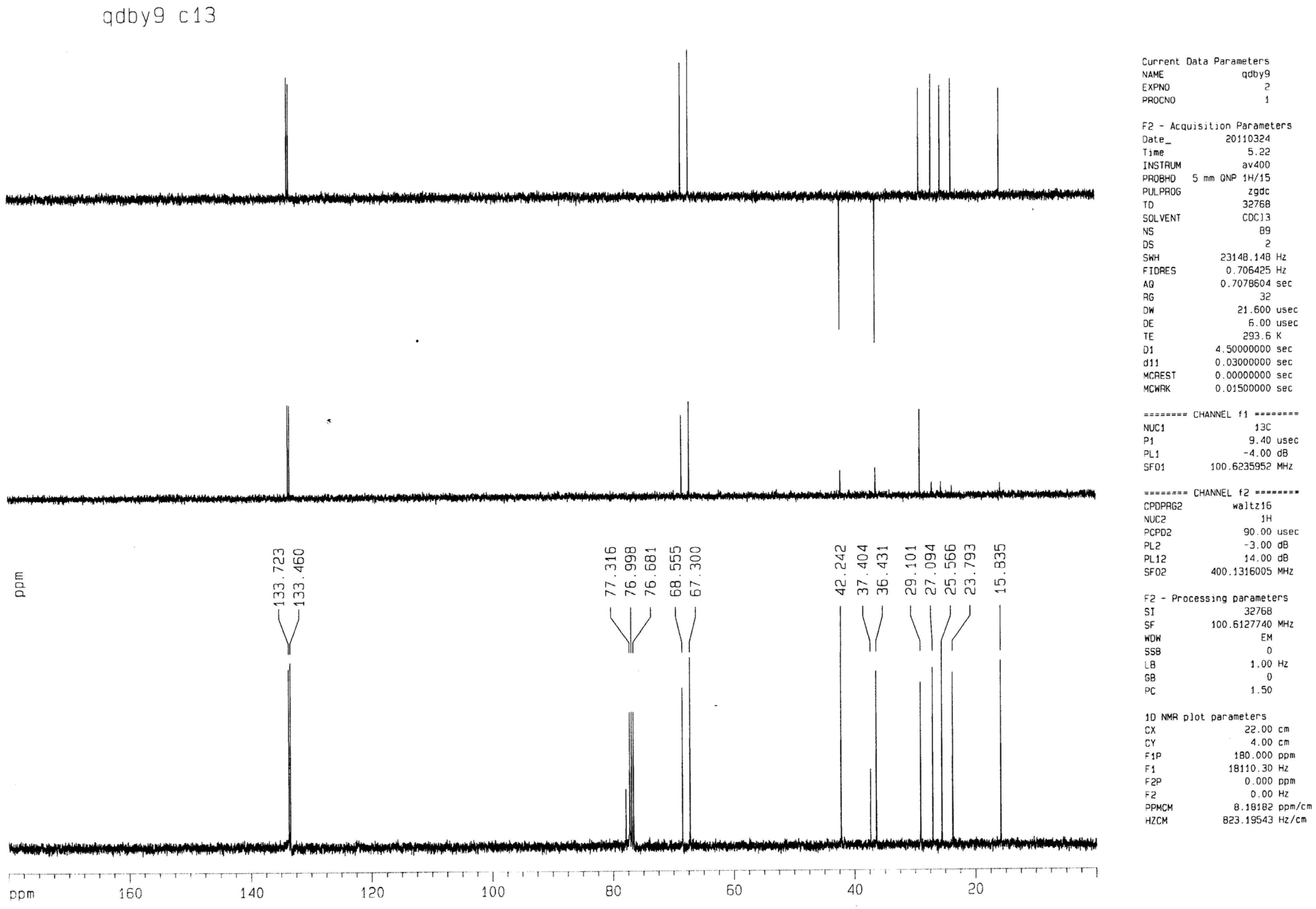

Figure 12. ^{13}C NMR spectrum of badounoid B (**2**)

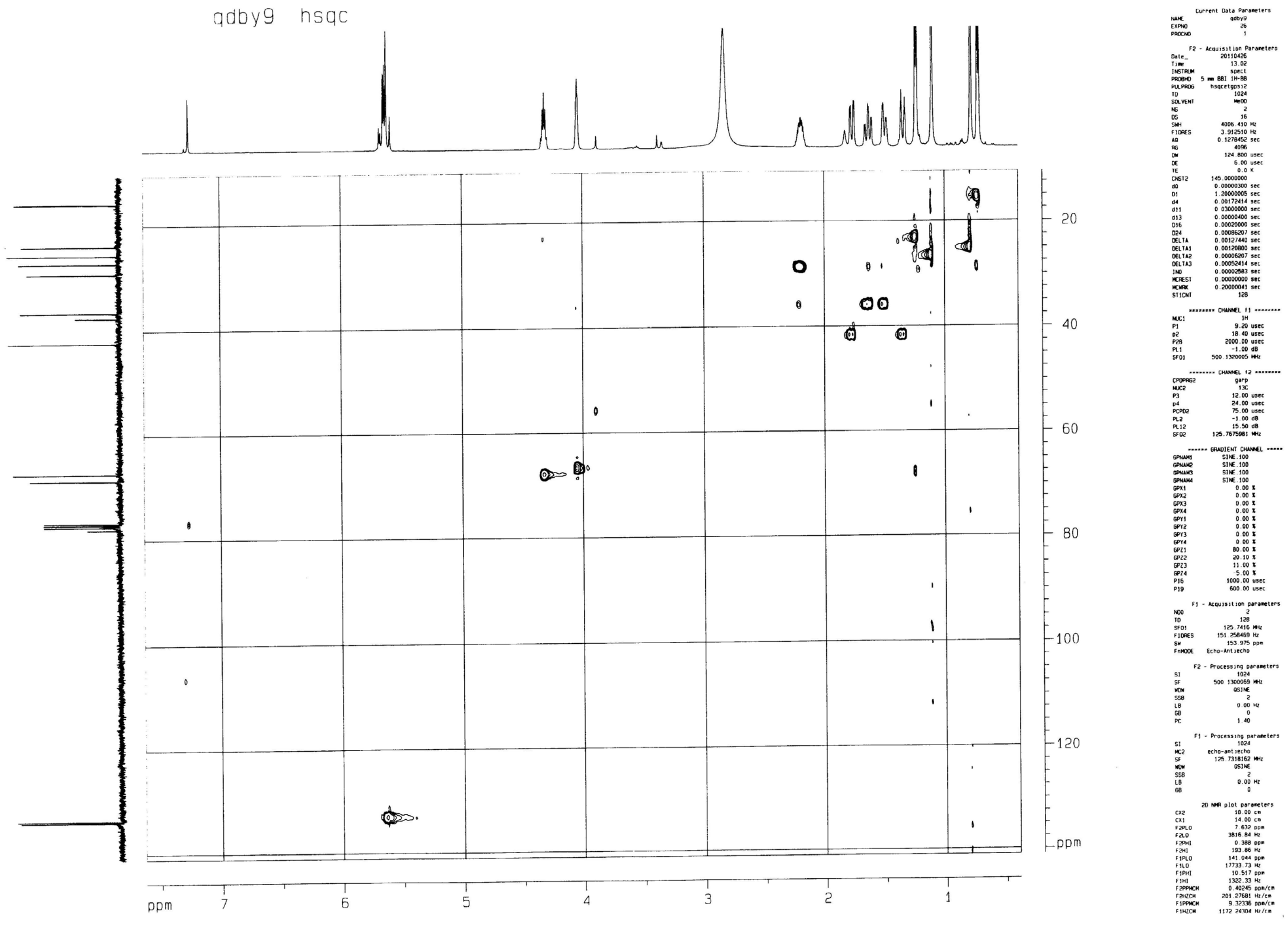

Figure 13. HSQC spectrum of badounoid B (**2**)

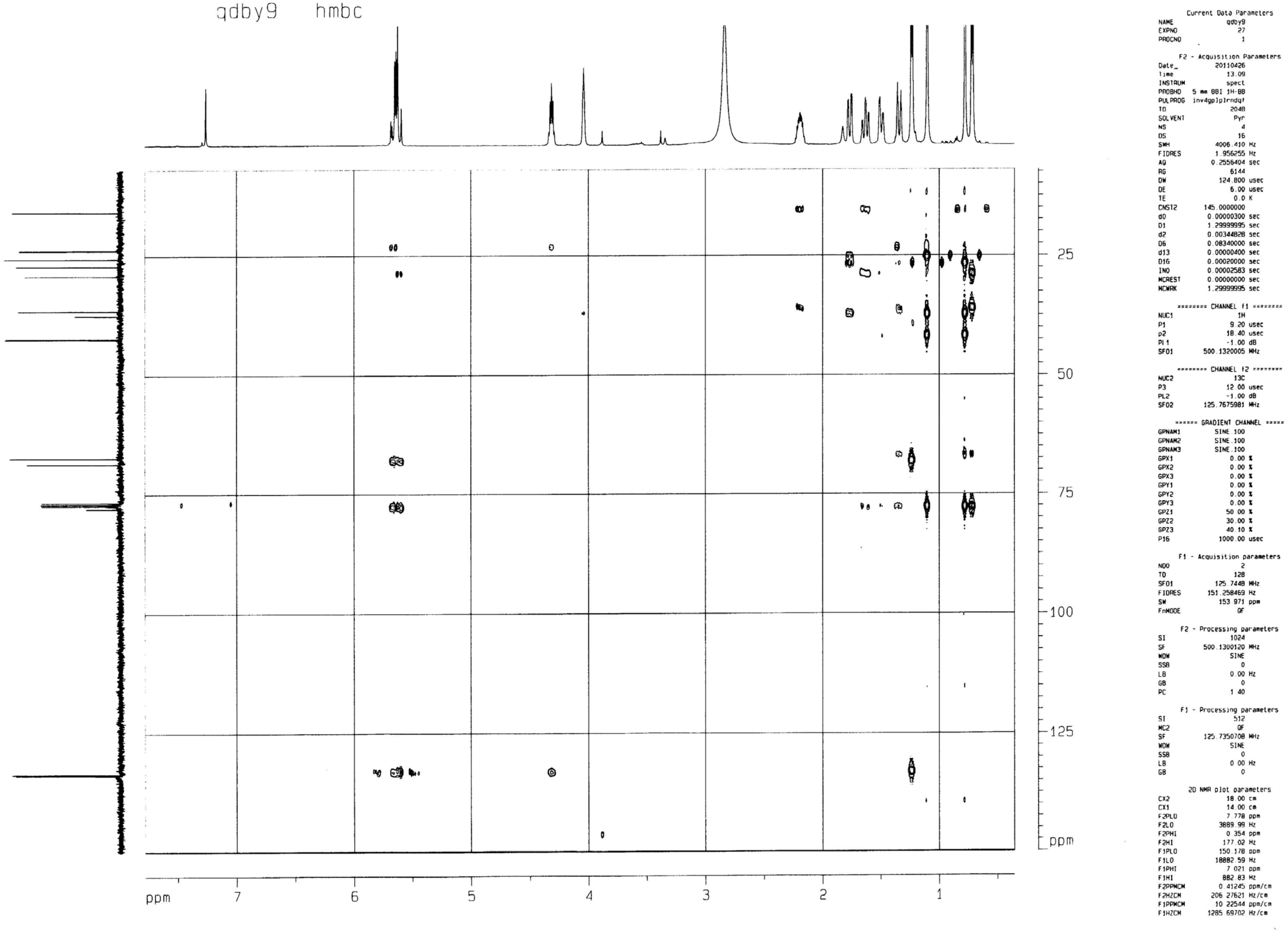

Figure 14. HMBC spectrum of badounoid B (**2**)

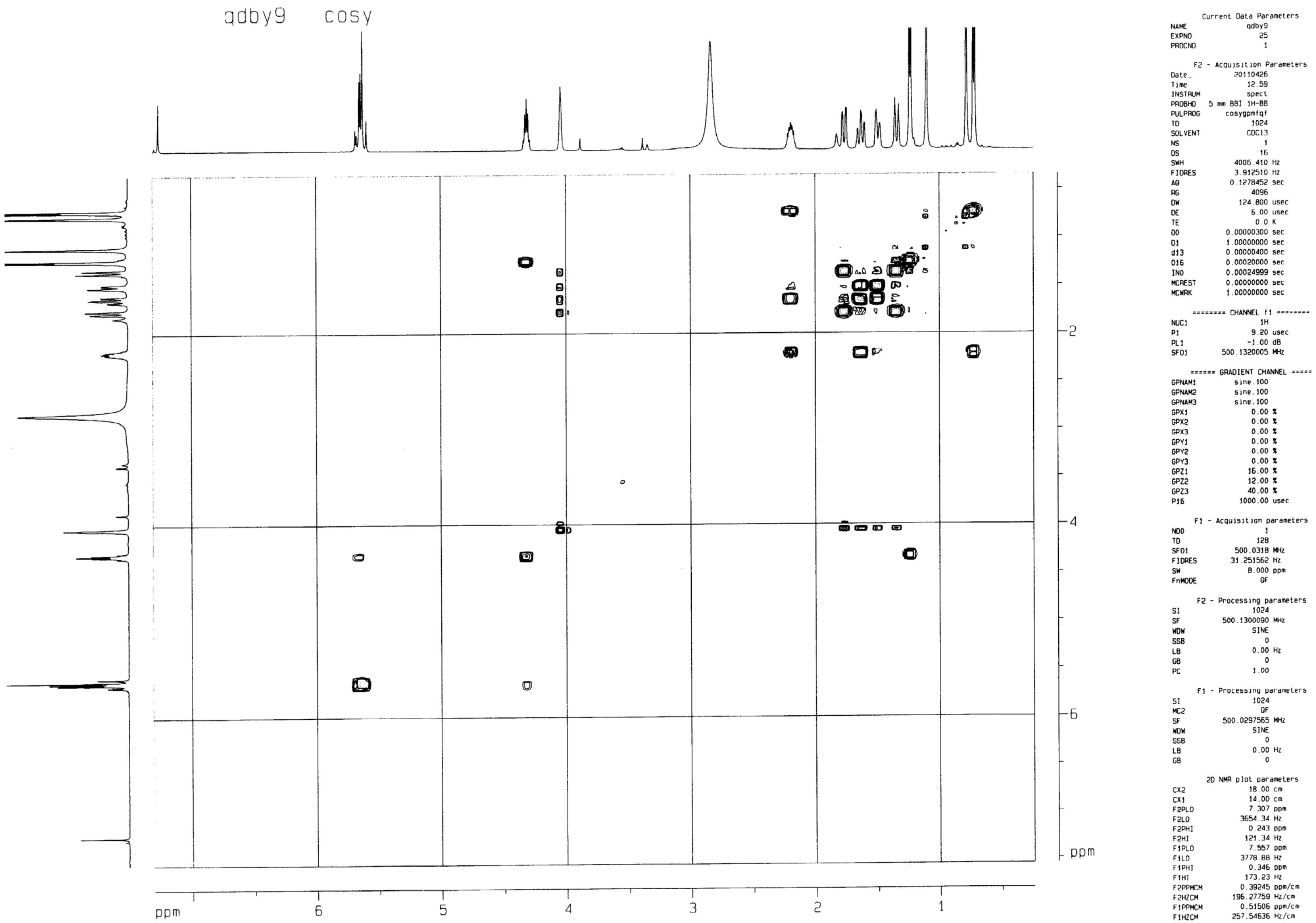

Figure 15. COSY spectrum of badounoid B (**2**)

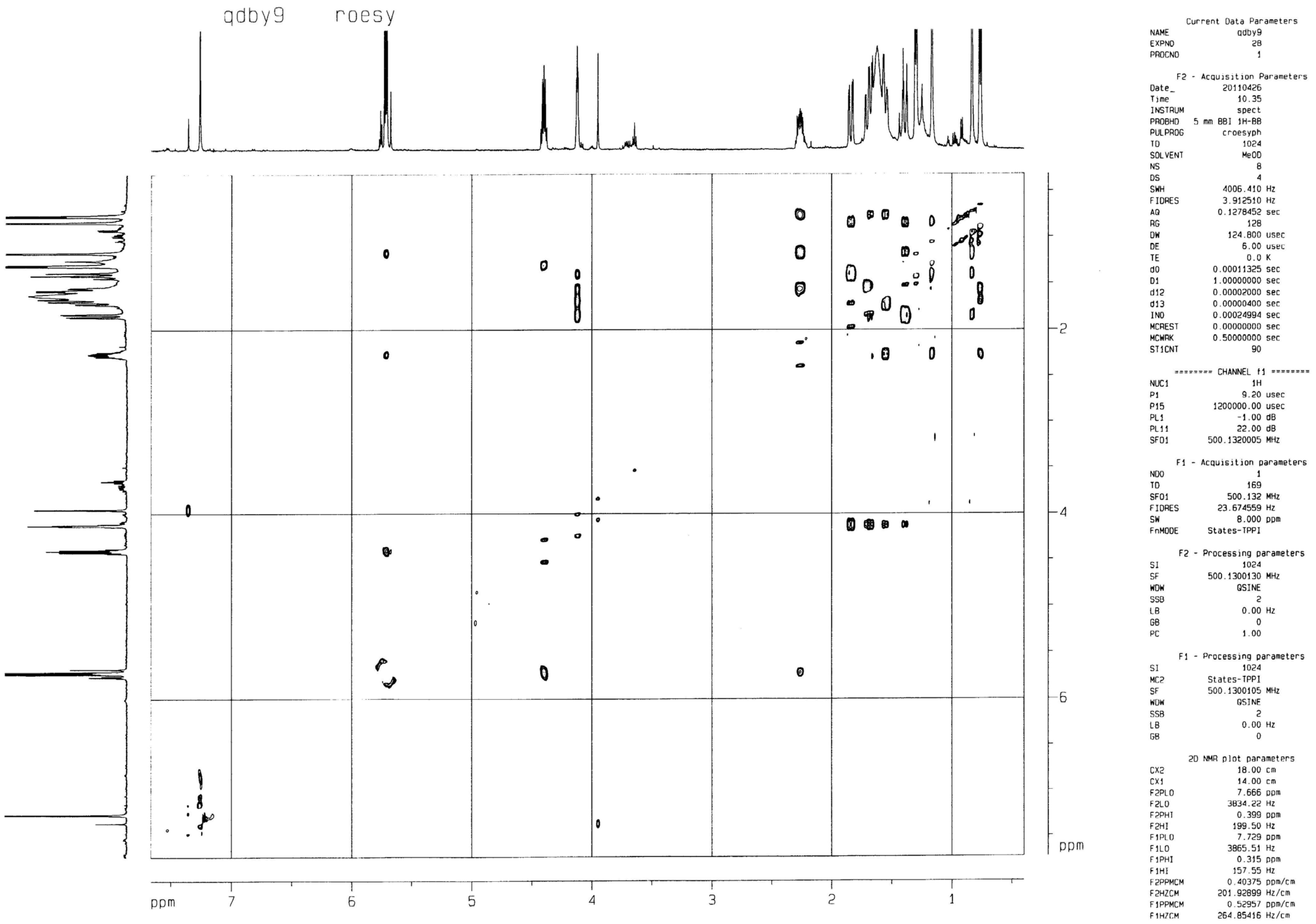

Figure 16. ROESY spectrum of badounoid B (**2**)

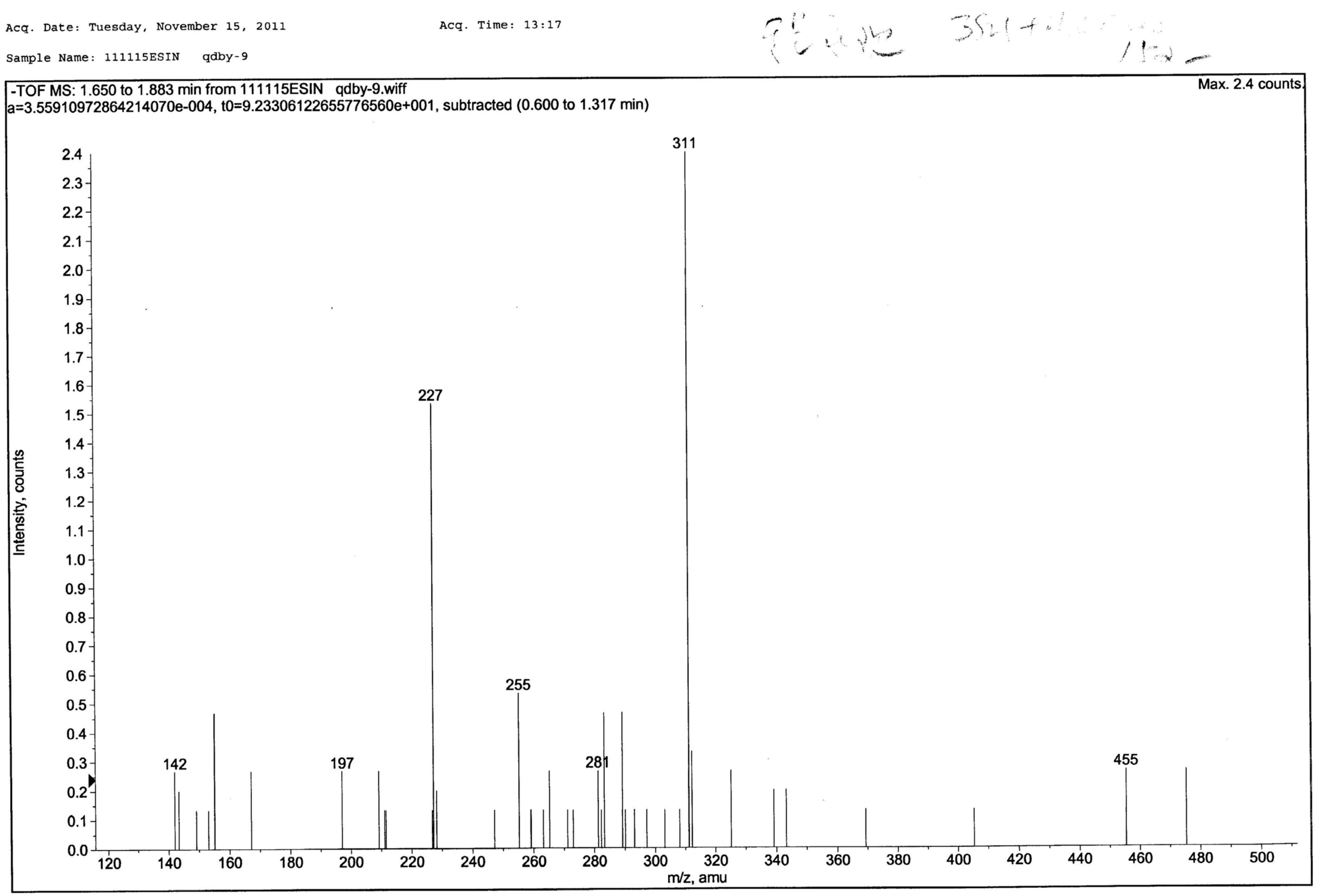

Figure 17. ESI-MS spectrum of badounoid B (**2**)

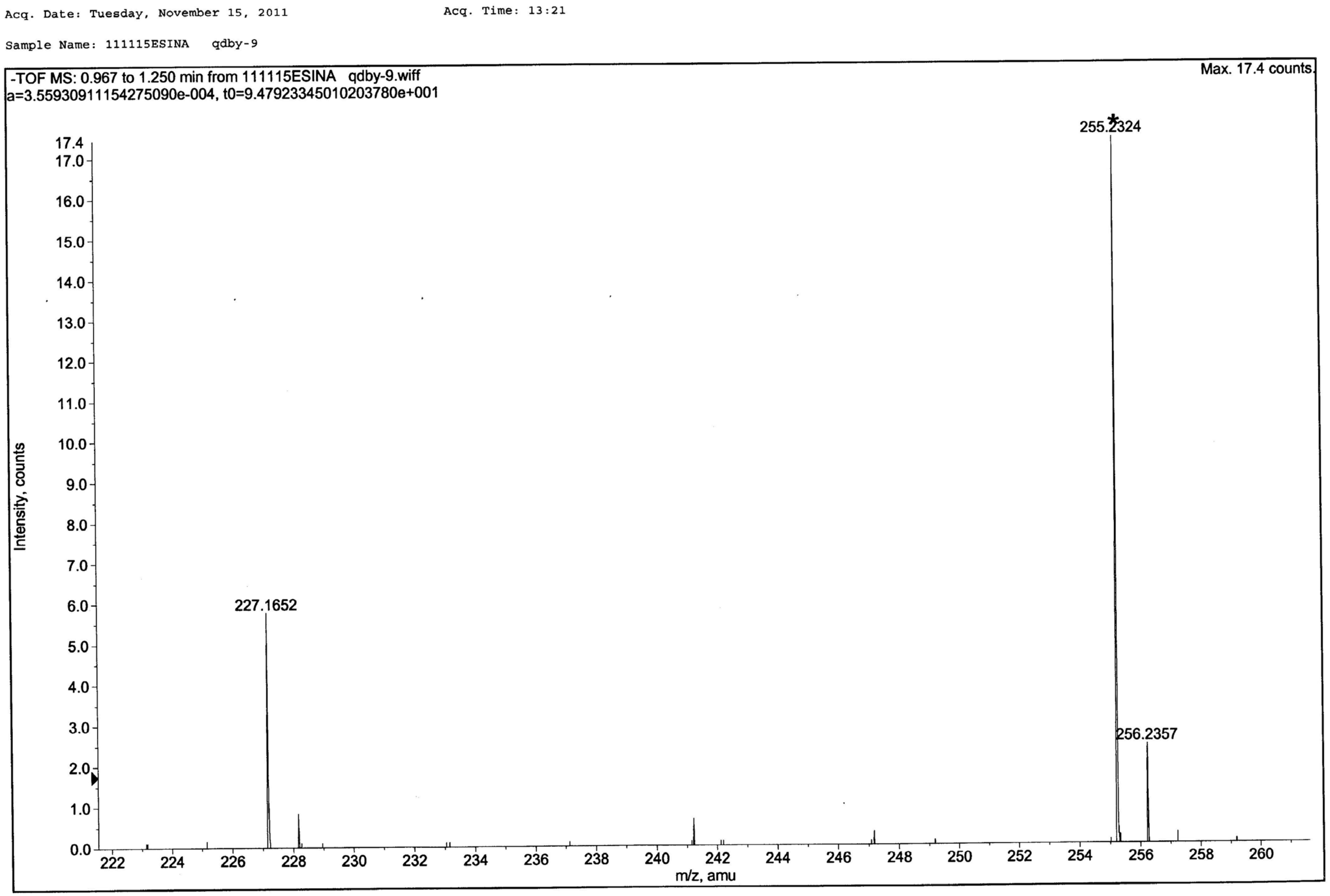

Figure 18. HRESI-MS spectrum of badounoid B (**2**)

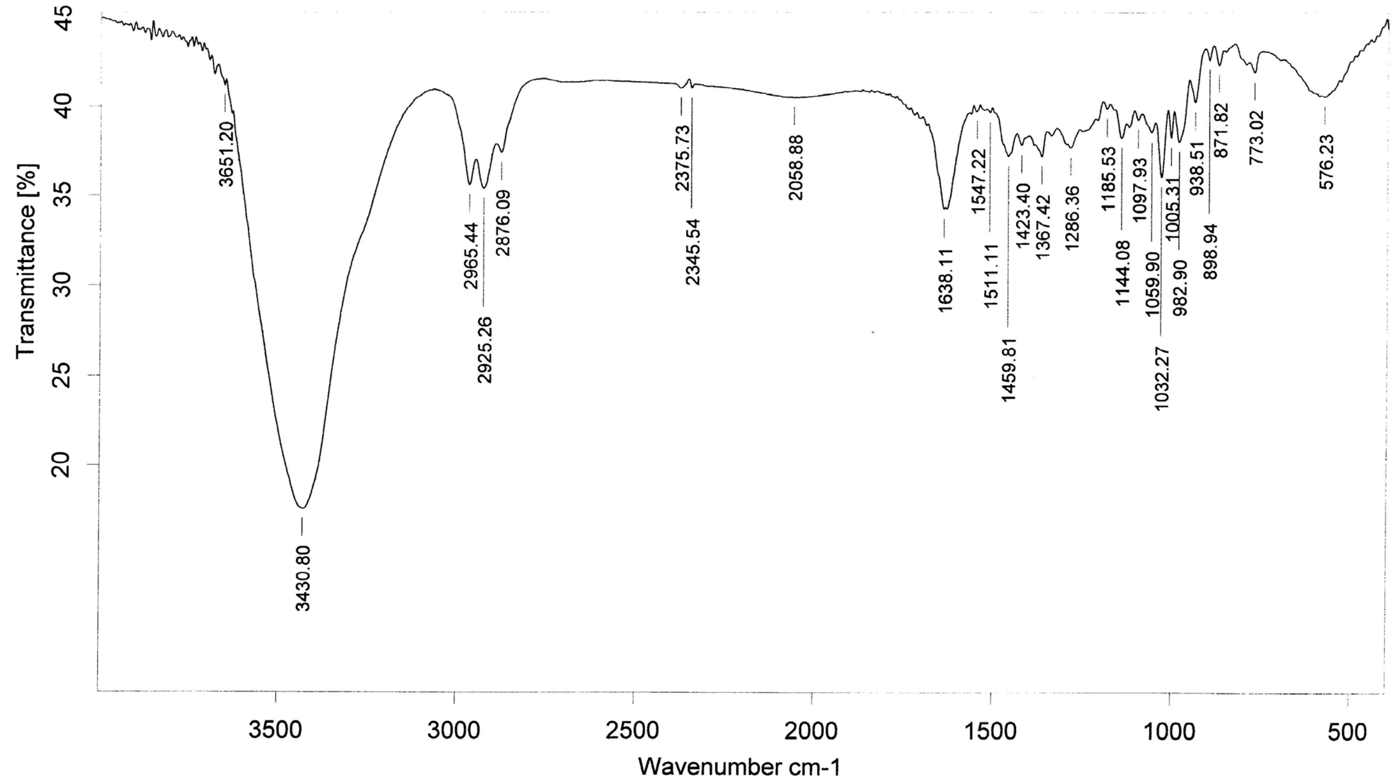

Sample : QDBY-9	Frequency Range : 399.246 - 3996.32		Measured on : 17/11/2011
Technique : KBr压片	Resolution : 4	Instrument : Tensor27	Sample Scans : 16
Customer : 111117IR1	Zerofilling : 2	Acquisition : Double Sided,For	

Figure 19. IR spectrum of badounoid B (**2**)

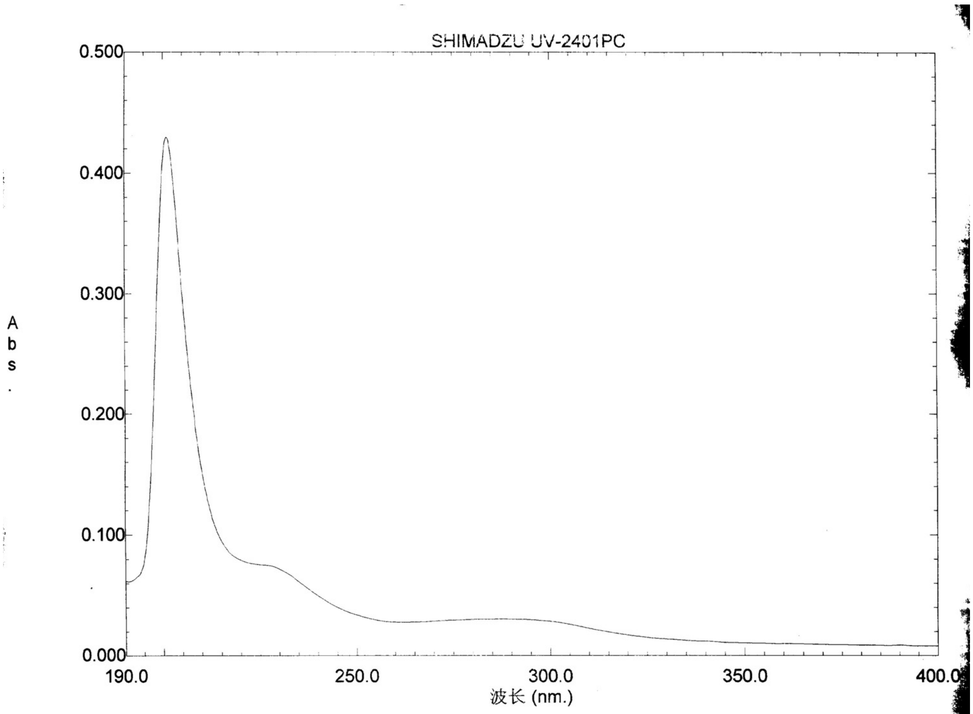

文件名: QDBY-9

QDBY-9 ———

样品浓度：0.0322毫克/毫升
溶剂：甲醇

创建于: 00:43 11-07-28
数据: 原始

测量模式: Abs.
扫描速度: 中速
狭缝: 5.0
采样间隔: 0.2

否.	波长 (nm.)	Abs.
1	286.60	0.0304
2	201.00	0.4296

Figure 20. UV spectrum of badounoid B (**2**)
